# Metal resistance or tolerance? Acidophiles confront high metal loads via both abiotic and biotic mechanisms

**DOI:** 10.3389/fmicb.2014.00157

**Published:** 2014-04-09

**Authors:** Mark Dopson, Francisco J. Ossandon, Lars Lövgren, David S. Holmes

**Affiliations:** ^1^Department of Biology and Environmental Sciences and Centre for Ecology and Evolution in Microbial Model Systems, Linnaeus UniversityKalmar, Sweden; ^2^Center for Bioinformatics and Genome Biology, Fundacion Ciencia y Vida and Departamento Ciencias Biologicas, Facultad de Ciencias Biológicas, Universidad Andrés BelloSantiago, Chile; ^3^Department of Chemistry, Umeå UniversityUmeå, Sweden

**Keywords:** metal, acidophile, resistance, tolerance, homeostasis, biomining

## Abstract

All metals are toxic at high concentrations and consequently their intracellular concentrations must be regulated. Extremely acidophilic microorganisms have an optimum growth of pH <3 and proliferate in natural and anthropogenic low pH environments. Some acidophiles are involved in the catalysis of sulfide mineral dissolution, resulting in high concentrations of metals in solution. Acidophiles are often described as highly metal resistant via mechanisms such as multiple and/or more efficient active resistance systems than are present in neutrophiles. However, this is not the case for all acidophiles and we contend that their growth in high metal concentrations is partially due to an intrinsic tolerance as a consequence of the environment in which they live. In this perspective, we highlight metal tolerance via complexation of free metals by sulfate ions and passive tolerance to metal influx via an internal positive cytoplasmic transmembrane potential. These tolerance mechanisms have been largely ignored in past studies of acidophile growth in the presence of metals and should be taken into account.

## INTRODUCTION

Microorganisms utilize metals as structural components of biomolecules, as cofactors in reversible oxidation/reduction reactions and in electron transfer chains during energy conservation. However, metals can become toxic if their intracellular concentrations are too high. Therefore, metal (and metalloid) homeostasis and resistance systems are required to maintain optimal intracellular metal concentrations (Nies and Silver, 2007).

Acidophilic microorganisms (optimal growth pH <3) often grow in metal rich environments such as acid sulfate soils containing iron sulfides (Wu et al., 2013) and milieus associated with metal sulfide mining (Dopson and Johnson, 2012). As many metals are more soluble at acidic pH, acidophiles are typically exposed to high metal concentrations and can survive in ≤1000-fold higher amounts than neutrophilic microorganisms (Dopson et al., 2003). As a consequence, they are often described as highly metal resistant and that they have multiple and/or more efficient active resistance systems than are present in neutrophiles. However, some acidophiles do not appear to have more metal resistance genes and we contend that their growth in high metal concentrations is partially due to an intrinsic tolerance as a consequence of the environment in which they live.

## ACIDOPHILE METAL RESISTANCE OR TOLERANCE?

Acidophile metal resistance strategies do not fully explain why they are able to grow in solution with very high concentrations of metals. Below we describe largely ignored acidophile metal tolerance systems such as complexation of free metals by sulfate ions and passive tolerance to metal influx via an internal positive cytoplasmic transmembrane potential.

### METAL SPECIATION AND ACIDOPHILES

**FIGURE 1:**
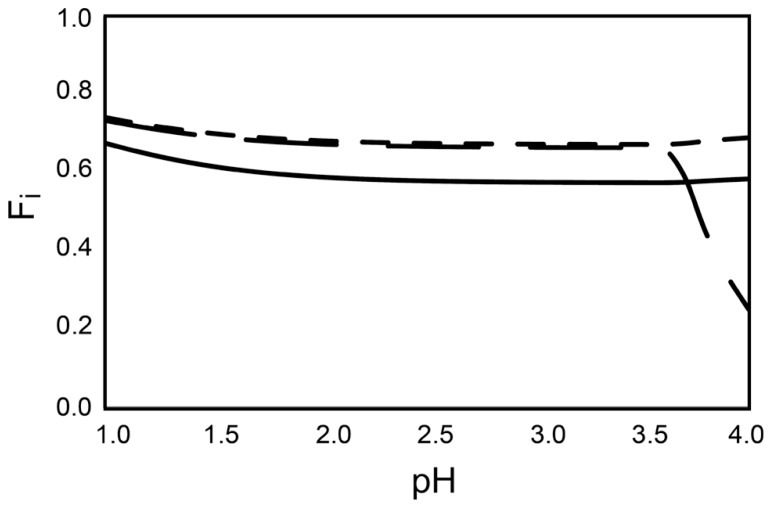
**The fraction of total free metal ion (Fi) for each of the metals.** The amount of free metal ion (Fi) of Zn^2+^ (solid line), Cu^2+^ (long dashes), and Ni^2+^ (short dashes) denoted as a fraction (100% free ion defined as 1.0) of total metal ion present in each of the species. The metal concentrations are calculated for systems with 200 mM of the respective metals, 285 mM SO_4_^2-^, and 50 mM Fe^2+^. The calculations are made using the computer code WinSGW (Karlsson and Lindgren, 2006) based on the speciation code Solgaswater (Eriksson, 1979). Equilibrium constants are collected from the equilibrium database Hydra/Medusa (Puigdomenech, 2004).

**FIGURE 2:**
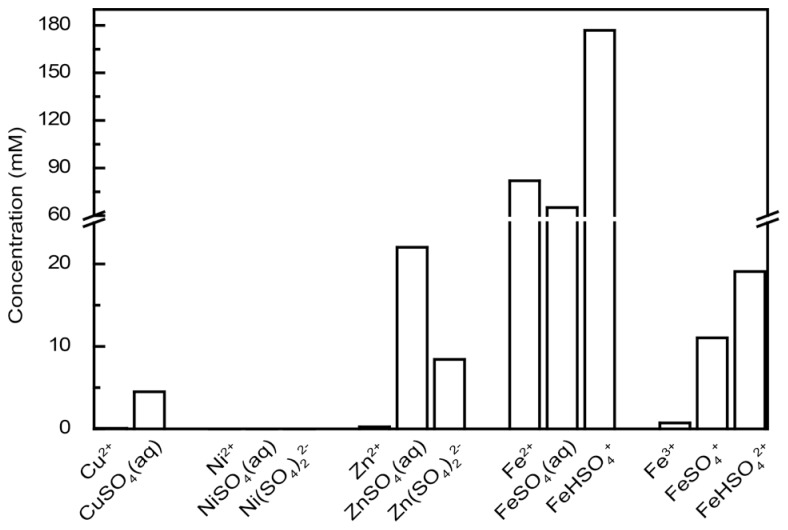
**The aqueous metal speciation in Iron Mountain, California.** The concentrations of free metal ions and metal speciation by sulfate are calculated assuming the composition of sample 90WA103 from the Richmond Mine, Iron Mountain (Nordstrom and Alpers, 1999) at pH 0.48. A temperature of 25°C is assumed in the calculations and other metal concentrations are considered to be zero.

In modern ecotoxicology, it is acknowledged that the distribution of metals between different chemical species (the speciation) must be accounted for when their ecotoxic effects are assessed (Christiansen et al., 2011) and that the free ion is the most toxic form of the metal (Di Toro et al., 2001). Acidophilic microorganisms often grow in environments containing high concentrations of sulfate ions that can complex metal cations at acidic pH. Therefore, the concentrations of free ions that can enter the cytoplasm and consequently challenge acidophiles are significantly lower than the total concentration of the metal. As a consequence, it is possible that extreme metal tolerance in acidophiles is partially a function of free metal ion complexation by sulfate that precludes the metal ion entry into the cell. The percentages of free metal ion for Cu^2+^, Ni^2+^, and Zn^2+^ were between 60 to 70% of the 200 mM metal ion calculated for each case (i.e., 60 to 80 mM of the metals were bound as sulfate ions and could not enter the cell; **Figure 1**). This was correct for a pH range from 1.0 to 3.5 that is typical for acidophilic microorganisms. When higher metal concentrations are present, the sulfate concentration would also likely be higher (metal sulfide dissolution also generates sulfate ions from oxidation of the sulfur moiety). An example calculated for an extreme acid mine drainage stream is at Iron Mountain, California that has 5 mM Cu, <1 mM Ni, 31 mM Zn, 324 mM Fe^2+^, and 39 mM Fe^3+^ that may be complexed by 1229 mM sulfate (Nordstrom and Alpers, 1999). Modeling of the metal speciation for this case showed >98% of all the metals other than Fe^2+^ were complexed by sulfate (**Figure 2**). Complexation of free metal by sulfate highlights the necessity of taking the speciation into account when metal tolerance is examined for acidophiles. This has been shown for Zn^2+^ toxicity to *Acidithiobacillus caldus*, *Acidimicrobium ferrooxidans*, and *Ferroplasma acidarmanus* (Mangold et al., 2012).

### METAL TOLERANCE CONFERRED BY pH HOMEOSTASIS AND pH OPTIMA

Cytoplasmic membranes have three electrostatic potentials: the transmembrane potential, the dipole potential, and the surface potential (Wang, 2012). Acidophiles have an inside positive cytoplasmic transmembrane potential (neutrophiles have an inside negative potential) that is reinforced by the dipole potential. This helps to maintain pH homeostasis as H^+^ must travel “up-hill” against a chemiosmotic gradient to enter the cytoplasm (Baker-Austin and Dopson, 2007; Slonczewski et al., 2009). The transmembrane and dipole potentials also provide a hindrance to metal cation influx. If this barrier is sufficiently strong then only a portion of the available free metal ion will penetrate the cell and cause a toxicity response. This potential method of intrinsic metal tolerance may have conferred the ability to withstand high metal concentrations before the evolution of more complex pumps.

A further effect of acidophile optimum growth pH is increased competition between cations and protons for cell surface binding sites (Heijerick et al., 2002). This is supported by growth experiments with *At. caldus* where Zn toxicity decreased at lower pH values (Stefanie Mangold, Ph.D. thesis, Umeå University), potentially as less metal enters the cytoplasm at acidic pH. The possibility that only a small fraction of extracellular metals cross the cell membrane is supported by the small increase in intracellular Zn observed in resting cells of *At. caldus*, *Ac. ferrooxidans*, and *F. acidarmanus* and a lack of a general stress response when these species are cultured with external concentrations up to 200 mM Zn (Mangold et al., 2012). However, it is not supported by the detection of stress proteins in the presence of more toxic metals such as Cu in *F. acidarmanus* (Baker-Austin et al., 2005) and *Metallosphaera sedula* (Maezato et al., 2012); Cu and Cd in *Sulfolobus metallicus* (Orell et al., 2013); Fe^2+^ in *F. acidarmanus* (Dopson et al., 2005); and in the natural Iron Mountain acid mine drainage community that is challenged by very high metal concentrations (Ram et al., 2005).

### HIGHER FREQUENCY OF KNOWN METAL RESISTANCE SYSTEMS

A further method by which acidophiles are more metal resistant is that their genomes contain genes encoding for more metal resistance systems than in neutrophiles. An example of a multiple metal resistance systems is the ATPase and Cus systems encoded on a gene island in *Acidithiobacillus ferrooxidans* ATCC 53993 that is not present on the type strain that may explain its higher Cu resistance (Orellana and Jerez, 2011). To test if acidophiles have a higher frequency of metal resistance systems, the percentage of genes related to Zn and Cu resistance within the total number of genes encoded in the respected genomes were separately calculated for 23 neutrophile and 21 acidophile genomes. The genomes were chosen based on three criteria: (1) the genome sequences were publicly available at the time that the analysis was initiated; (2) they were derived from different branches of the phylogenetic tree and include Bacteria and Archaea; and (3) the sequences were derived from organisms in which the associated metadata was available (e.g., phylogeny, temperature, and pH optima). The results for both Zn and Cu showed no significant difference calculated as the average percentage of genes ±SD for the acidophiles compared to the neutrophiles (0.28% ± 0.28 compared to 0.17% ± 0.14, respectively, for the zinc resistance gene analysis and 0.25% ± 0.23 compared to 0.15% ± 0.12, respectively, for copper resistance). A further pairwise *t*-test comparing Zn and Cu resistance genes between acidophiles and neutrophiles based upon the phylogeny of the species also showed no significant difference for both Zn (*p* = 0.49) and Cu (*p* = 0.37). Although the difference between the acidophiles and neutrophiles is insignificant, it is interesting that the 7 acidophile genomes with the highest percentage of Zn resistance genes include all of the analyzed *Acidithiobacillus* spp. and *Leptospirillum ferriphilum* that are commonly identified in biomining environments and therefore challenged by high metal concentrations (Okibe et al., 2003; Dopson and Lindström, 2004). However, not all common biomining microorganisms have higher levels of Zn resistance genes as the highly metal resistant species; *F. acidarmanus* (Dopson et al., 2004) only contains 0.05% of genes annotated as related to Zn resistance. A possibility is that species capable of growth at higher pH values, such as the *Acidithiobacillus* spp. (Mielke et al., 2003), have a lower competition between protons and cations for binding sites (discussed above) that is compensated for by more resistance genes. In support of this explanation is that the archaea, and in particular those species capable of growth close to pH 0, tend to have lower percentages of Zn resistance genes than bacteria. The higher number of acidophile genes related to Cu resistance may be due to its higher availability as a free ion at acidic pH compared to neutral conditions. It is not known if these trends also hold for other metals than Zn and Cu and further studies are needed.

### OXYANIONS, AN EXCEPTION TO THE RULE?

Arsenic is predominantly present in biomining environments as the metalloids, arsenate (AsO_4_^3-^) and arsenite (AsO_3_^3-^ at neutral pH and AsOH_3_ at acidic pH). Therefore, the intrinsic acidophile cation tolerance systems described in this review would not aid in arsenic resistance. This may at least partially explain why acidophiles are <100-fold more resistant to arsenic than neutrophiles when they are up to 1000-fold more resistant than neutrophiles to metal cations (Dopson et al., 2003). This would also be true for other oxyanions, such as for molybdenum and vanadium.

## CONCLUSION

It is very difficult to compare resistance between microbial species as metal toxicity is dependent on its biological availability (free ion toxicity), the solution chemistry, and the variable toxicity of metal ions to specific cellular functions. Acidophiles have a variety of intrinsic and active metal resistance systems that likely combine to permit their growth in very high metal concentrations. Also, it cannot be ruled out that novel, previously undetected resistance systems are present that contribute to active acidophile metal resistance. The potential contribution of abiotic factors such as metal speciation combined with metal tolerance afforded by the internal positive transmembrane and dipole potentials, and competition for binding sites to acidophile metal resistance has been largely overlooked. In the future, these factors should also be taken into account when assessing acidophile growth in high metal loads.

## Conflict of Interest Statement

The authors declare that the research was conducted in the absence of any commercial or financial relationships that could be construed as a potential conflict of interest.
